# Molecular subtypes based on N6-methyladenosine RNA methylation demonstrate the heterogeneity of immune and biological functions in pediatric septic shock

**DOI:** 10.1016/j.heliyon.2023.e20714

**Published:** 2023-10-06

**Authors:** Huabin Wang, Junbin Huang, Cheng Guo, Jingfang Wu, Liyuan Zhang, Xueyun Ren, Lijun Gan

**Affiliations:** a Department of Neonatal Intensive Care Unit, Affiliated Hospital of Jining Medical University, Jining Medical University, Jining 272000, China; b Postdoctoral Mobile Station of Shandong University of Traditional Chinese Medicine, Jinan 250000, China; c Department of Pediatrics, Affiliated Hospital of Jining Medical University, Jining Medical University, Jining 272000, China; d Division of Hematology/Oncology, Department of Pediatrics, the Seventh Affiliated Hospital of Sun Yat-Sen University, Shenzhen, 518107, China; e Department of Pediatric Intensive Care Unit, Affiliated Hospital of Jining Medical University, Jining Medical University, Jining 272000, China; f Department of Cardiology, Affiliated Hospital of Jining Medical University, Jining Medical University, Jining 272000, China; g Jining Key Laboratory for Diagnosis and Treatment of Cardiovascular Diseases, Jining 272000, China

**Keywords:** Pediatric septic shock, m6A RNA methylation, Risk prediction model, Molecular subtype

## Abstract

**Introduction:**

Septic shock in children is a highly heterogeneous syndrome involving different immune states and biological processes. We used a bioinformatics approach to explore the relationship between N6-methyladenosine (m6A) methylation and septic shock in children.

**Methods:**

A gene expression dataset including information on 98 children with septic shock was selected. To construct and evaluate a risk prediction model, machine learning was used to screen marker m6A regulators. Based on differentially expressed m6A regulators, molecular subtypes for paediatric septic shock were constructed. Subsequently, the differences in the m6Ascore, heterogeneity of immune cell infiltration, and heterogeneity of biological functions between the different subtypes were analyzed. Finally, real-time quantitative PCR (RT-qPCR) was performed to validate the expression of the marker m6A regulators.

**Results:**

Fifteen differentially expressed m6A regulators were identified. Six marker m6A regulators, including LRPPRC, ELAVL1, RBM15, CBLL1, FTO, and RBM15B, were screened using the random forest method. The risk prediction model for paediatric septic shock constructed using m6A markers had strong consistency and high clinical practicability. Two subtypes of paediatric septic shock have been identified based on the differential expression pattern of m6A regulators. Significant differences were observed in RNA epigenetics, immune statuses, and biological processes between the two m6A subtypes. Differentially expressed genes between the two subtypes were enriched in cell number homeostasis, redox responses, and innate immune system responses. Finally, the six marker m6A regulators were verified in additional samples.

**Conclusions:**

Based on the heterogeneity of m6A methylation-regulated genes, two different subtypes of septic shock in children with different RNA epigenetics, immune statuses, and biological processes were identified, revealing the heterogeneity of the disease largely attributable to differential m6A methylation. The findings will help explore and establish appropriate individualized treatments.

## Highlights

• This study is the first to analyse m6A levels in paediatric septic shock.
• LRPPRC, ELAVL1, RBM15, CBLL1, FTO and RBM15B were marker m6A regulators.
• The risk prediction model had strong consistency and high clinical utility.
• Molecular subtypes demonstrated heterogeneity in immune and biological functions.

## 1 Introduction

Sepsis is a common critical illness in paediatric patients. Sepsis is caused by the dysregulated response of an organism to infection, leading to microcirculatory disturbances, immune dysfunction, and organ dysfunction. Severe illness can lead to life-threatening septic shock [1]. Of the approximately 7.6 million annual deaths in children younger than 5 years, 64 % are related to sepsis or septic shock caused by severe infection [2,3]. Mortality rates can reach up to 20–50 % in children with septic shock treated in paediatric intensive care units (PICUs) [[4], [5], [6]]. Failure of treatment regimens for septic shock in children is largely attributed to the disease heterogeneity and the inability to accurately classify patients at the molecular level. Clarification of different subtypes of septic shock and appropriate treatment regimens can improve the survival rate of children with septic shock [7,8].

N6-methyladenosine (m6A) is a common form of RNA methylation in eukaryotes and is involved in many important physiological processes, including immunity, metabolism, proliferation, and DNA damage responses, particularly in immune inflammatory reactions [[9], [10], [11]]. Therefore, the heterogeneity of septic shock in children may be related to m6A RNA modifications. m6A methylation is a reversible biological process owing to its dynamic regulation by methyltransferases, demethylases, and binding proteins. m6A has been widely studied as a potential therapeutic target for a variety of diseases [[12], [13], [14]]. Zhang et al. found that knocking down METTL3 inhibits osteoblast differentiation and Smad-dependent signalling by stabilising Smad7 and Smurf1 mRNA transcripts through YTHDF2 involvement and activates inflammatory responses by regulating MAPK signalling in lipopolysaccharide (LPS)-induced inflammation [15]. In another study, upon stimulation with LPS, mettl3-deficient macrophages exhibited reduced production of inflammatory factors. Consistently, Mettl3^flox/flox^; Lyzm-Cre mice displayed increased susceptibility to bacterial infection and faster tumour growth. Owing to technical limitations, only one or two m6A regulators and cell types were investigated. The anti-inflammatory effect is characterised by interactions between numerous immune cells and immune regulatory factors in a highly coordinated manner [16]. Therefore, a comprehensive understanding of the immune microenvironment mediated by multiple m6A regulators is expected to deepen our understanding of m6A immune regulation. However, the specific effects of the m6A regulators on the heterogeneity of paediatric septic shock remain largely unknown. Studying the role of m6A methylation modification in children with septic shock and clarifying whether it plays a central role in the occurrence and development of the disease will help identify m6A regulators as candidate diagnostic and treatment targets.

In this study, we systematically analyzed the expression signatures of widely reported m6A RNA methylation regulators based on gene sets from the Gene Expression Omnibus (GEO) database of paediatric septic shock and elaborated on the heterogeneity of the disease by constructing m6A molecular subtypes.

## 2 Materials and methods

### 2.1 Data downloading and preprocessing

The peripheral blood transcriptome dataset, GSE26440 for paediatric septic shock was obtained from the GEO database [17,18]. Series matrix files (txt format) and annotation files of GPL570 (HG-U133_Plus_2; Affymetrix Human Genome U133 Plus 2.0 Array) were downloaded. The GSE26440 dataset contained the transcriptome data (mRNA) of 98 children with septic shock and 32 normal controls. Genes were annotated using Perl v5.30.0, and data were normalised using the limma package in R v4.1.0. Finally, an expression matrix was obtained for the analysis.

### 2.2 Differential expression analysis and correlation analysis of m6A regulators

The mRNA expression levels of the 24 m6A regulators in all samples were determined. These 24 m6A regulators included eight writers (METTL3, METTL14, KIAA1429, WTAP, ZC3H13, RBM15, RBM15B, and CBLL1), two erasers (ALKBH5 and FTO), and 14 readers (YTHDC1, YTHDC2, YTHDF1, YTHDF2, YTHDF3, HNRNPC, FMR1, LRPPRC, HNRNPA2B1, IGFBP1, IGFBP2, IGFBP3, ELAVL1, and IGF2BP1) (Table 1). Differential expression of m6A regulators was compared between the septic shock and control groups. Heatmaps and boxplots were generated using the pheatmap and ggboxplot packages.

**Table 1:** Twenty-four m6A regulators were analyzed in this study.

Gene	Type
METTL3	writers
METTL14	writers
KIAA1429	writers
WTAP	writers
ZC3H13	writers
RBM15	writers
RBM15B	writers
CBLL1	writers
YTHDC1	readers
YTHDC2	readers
YTHDF1	readers
YTHDF2	readers
YTHDF3	readers
HNRNPC	readers
FMR1	readers
LRPPRC	readers
HNRNPA2B1	readers
IGFBP1	readers
IGFBP2	readers
IGFBP3	readers
ELAVL1	readers
IGF2BP1	readers
FTO	erasers
ALKBH5	erasers

Spearman's correlation was used to analyse the correlation between differentially expressed m6A regulators. A correlation analysis graph was generated using the CorrPlot package. The screening criteria were a correlation coefficient |r| > 0.3 and *p* < 0.001.

### 2.3 Screening marker m6A regulators

Among the differentially expressed m6A regulators, m6A genes were screened using the random forest (RF) method and support vector machine (SVM) algorithm [19,20]. A more suitable method for screening marker m6A regulators was selected from RF and SVM by plotting boxplots of residuals, inverse cumulative distributions of residuals, and receiver operating characteristic (ROC) curves. A low residual value and a high area under the curve (AUC) are more suitable for screening m6A regulators.

### 2.4 Constructing a risk prediction model for children with septic shock

The risk-prediction model for children with septic shock was established using the R package rms and visualised in the form of a nomogram. In the nomogram prediction model, the value of each independent variable was assigned according to its contribution to the independent variable to the outcome variable (i.e. the size of the regression coefficient). The scores for each independent variable were added to obtain the total score. Finally, the relationship between the total score and the probability of occurrence of the outcome event was established through a function transformation to calculate the probability of occurrence of the outcome event for the individual.

Calibration curves were constructed to assess the consistency of the prediction models. The calibration curve was a scatter diagram of the actual and predicted incidences. We used the lrm function in the rms R package to fit the logistic model and then used the calibration function to fit the calibration curve. The closer the fitted curve is to the reference line, the higher the calibration degree of the prediction model.

The clinical usefulness of the prediction model was evaluated using decision curve analysis (DCA) curves and clinical impact curves. The DCA and clinical impact curves were drawn using the rmda R package. A DCA curve was used to quantify the net benefit under different threshold probabilities to evaluate the clinical effectiveness of the prediction model. In the DCA curve, the abscissa is the threshold probability and the ordinate is the net benefit. If the DCA curve is very close to the two extreme curves, the clinical application value is small. If the net benefit rate of the DCA curve is higher than that of the extreme curve within a large abscissa range, the prediction model has a high application value.

The plot_clinical_impact function was used to plot the clinical impact curve of the model. The clinical impact curve shows the estimated number of individuals who would be at high risk for each risk threshold and visually shows the proportion of those cases (true positives). Of the 1000 patients, the red line shows the total number deemed at high risk for each risk threshold, and the blue line shows the number of true positives (cases). The closer the two curves are, the better the model's performance.

### 2.5 Exploring m6A molecular subtypes in paediatric septic shock

Consensus clustering and molecular subtype screening of GSE26640 were performed based on the expression profiles of the differentially expressed m6A regulators. The R package consensusclusterplus was used to classify all septic shock samples into k subtypes (k = 2–6). The k-means clustering algorithm was used for clustering. This method should provide the number of clusters and initialise the cluster centres (one for each cluster). We then calculated the distance from each point to each centroid and assigned the point to the centroid by measuring the shortest distance. Therefore, the new centroid was calculated from the newly allocated point, and the iteration continued until a balance was reached (no change in the assignment of new points) [21]. The optimal number of clusters was determined by the discriminatory clarity of the consensus matrix heatmap, the clustering score from the cumulative distribution function (CDF) curve, and the relative change in the area under the CDF curve. Principal component analysis (PCA) can reduce multiple variables to a set of principal components representing variables, that is, it converts high-dimensional data into low-dimensional data. We then verified the reliability of the consensus clustering using PCA cluster plots.

Based on the mRNA expression of m6A regulators with differential expression, we quantified the m6A modification pattern of each sample by PCA, resulting in an m6Ascore for each sample. We then compared the differences in the m6Ascore between the m6A subtypes.

### 2.6 Evaluation of the heterogeneity of immune cell infiltration between subtypes

First, we downloaded the immune gene dataset in the GMT format. Single-sample gene set enrichment analysis (ssGSEA) was performed using the GSVA and GSEA base packages to obtain the enrichment score (ES) of various types of immune cells in each sample, and the differences in immune cell infiltration levels between subtypes are shown as boxplots. Spearman's correlation was used to evaluate the correlation between the expression of m6A regulators and the degree of immune cell infiltration. The m6A regulators with the strongest correlation with immune cells were selected. The samples were divided into high and low-expression groups according to the median expression level of the m6A regulator, and the differences in the levels of immune cell infiltration between the two groups were subsequently compared.

### 2.7 Evaluation of heterogeneity in biological function between subtypes

Differentially expressed genes (DEGs) between different subtypes were identified using a linear model of the limma package. The screening criteria for DEGs were adjusted *p*-value <0.05, and |fold change (FC)| > 1.5. DEGs were subjected to Gene Ontology (GO) and Kyoto Encyclopedia of Genes and Genomes (KEGG) enrichment analyses using ClusterProfiler.

The gene expression matrix was subjected to Gene Set Enrichment Analysis (GSEA). “c2.cp.v7.2.symbols.gmt” was selected as the reference gene set. False discovery rate (FDR) (*q*-value) < 0.25 and adjusted *p*-value <0.05 showed statistically significant enrichment.

### 2.8 Expression of six marker m6A regulators in the validation dataset

The GSE26378 dataset containing the transcriptome data (mRNA) of 82 children with septic shock and 21 normal controls, was used to validate the expression of six marker m6A regulators [18].

### 2.9 Validation of six marker m6A regulators in the clinical cohort by real-time quantitative PCR (RT-qPCR)

Samples were collected from 12 healthy controls and 12 patients with septic shock who were hospitalised in the PICU of the Affiliated Hospital of Jining Medical University between January 1, 2023 and February 28, 2023. This study was approved by the Ethics Committee of the Affiliated Hospital of Jining Medical University (approval number: 2022C241). Consent was obtained from the guardians before the commencement of the study. The inclusion criteria were as follows: age, 1 month to 14 years; children who met the diagnostic criteria for septic shock and were hospitalised longer than 24 h. The exclusion criteria were malignant tumours, autoimmune diseases, and the use of immunosuppressive agents in the past 2 weeks.

Peripheral blood was collected from the patients on the first day of admission. Total RNA was extracted from whole blood using the FastPure Cell/Tissue Total RNA Isolation Kit V2 (Vazyme, RC112-01) following the manufacturer's instructions. Reverse transcription was performed using the HiScript III RT SuperMix for qPCR (Vazyme, R323-01). RT-qPCR was performed using the ChamQ Universal SYBR qPCR Master Mix (Vazyme, Q711-02). GAPDH was used as the reference gene. The relative expression levels were calculated using the 2^−ΔΔCT^ method. Table S2 lists the primer pairs used in the experiment.

### 2.10 Validation of six marker m6A regulators in RAW 264.7 cells and THP-1 cells by RT-qPCR

RAW 264.7 (a mouse-derived macrophage cell line) and THP-1 (a human monocytic leukemia cell line) cells were purchased from Procell Life Science Technology Co. Ltd. (Wuhan, China). Cells were cultured at 37 °C with 5 % CO_2._ The culture medium for RAW 264.7 cells was DMEM supplemented with 10 % FBS and 1 % penicillin-streptomycin. RAW 264.7 cells were seeded in a complete culture medium in 6-well plates at a density of 5 × 10^5^ cells/well. After overnight incubation, cells were treated with LPS (Solarbio, L8880) (1 μg/mL) for 24 h.

The culture medium for THP-1 cells was RPMI 1640 supplemented with 10 % FBS, 1 % penicillin-streptomycin, and 0.05 mM β-mercaptoethanol. THP-1 cells were also seeded in 6-well plates at a density of 5 × 10^5^ cells/well. THP-1 cells were differentiated into macrophages by incubating with 100 ng/mL phorbol 12-myristate 13-acetate (MultiSciences, CS0001) for 48 h. Then, cells were treated with LPS (1 μg/mL) for 24 h.

The other RT-qPCR steps were the same as those used in the clinical cohort. Table S2 lists the primer pairs used in the experiment.

### 2.11 Statistical analysis

All statistical analyses were performed using the R software, version 4.1.0. Multiple testing corrections were performed using Benjamini-Hochberg method, and FDR correction was performed for multiple testing to reduce the rate of false positives. For two groups of continuous variables, differences between normally distributed variables were compared using the Student's t-test, and those between non-normally distributed variables were compared using the Mann-Whitney *U* test. All *p* values were two-sided, and *p* < 0.05 was considered statistically significant.

## 3 Results

### 3.1 Characteristics of datasets and patients

Expression data from 98 children with septic shock and 32 healthy controls were generated using whole blood-derived RNA samples representing the parameters in the first 24 h of admission to the PICU at the Cincinnati Children's Hospital Medical Center. There were 71 males (54.6 %) in the entire dataset; the median age was 24 months. Among the infection characteristics of paediatric patients with septic shock, 23 cases of gram-positive bacteria (23.5 %), 20 cases of gram-negative bacteria (20.4 %), and 45 cases of negative cultures (45.9 %) were noted. Further information on the dataset is presented in Table S1.

### 3.2 Differential expression analysis and correlation analysis for m6A regulators

**Fig. 1:**
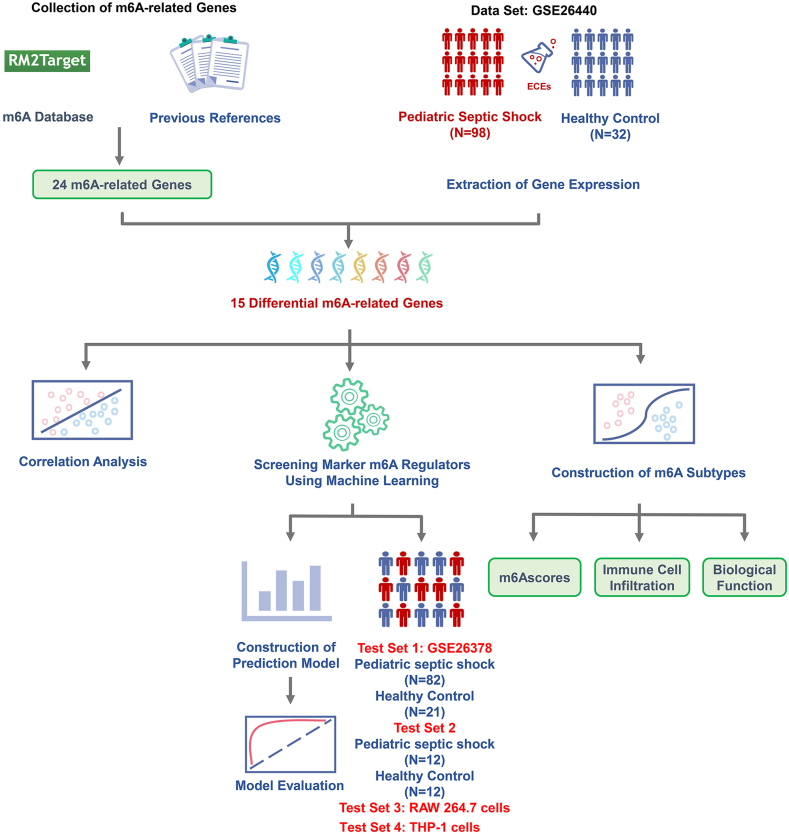
Flow chart of the study.

**Fig. 2:**
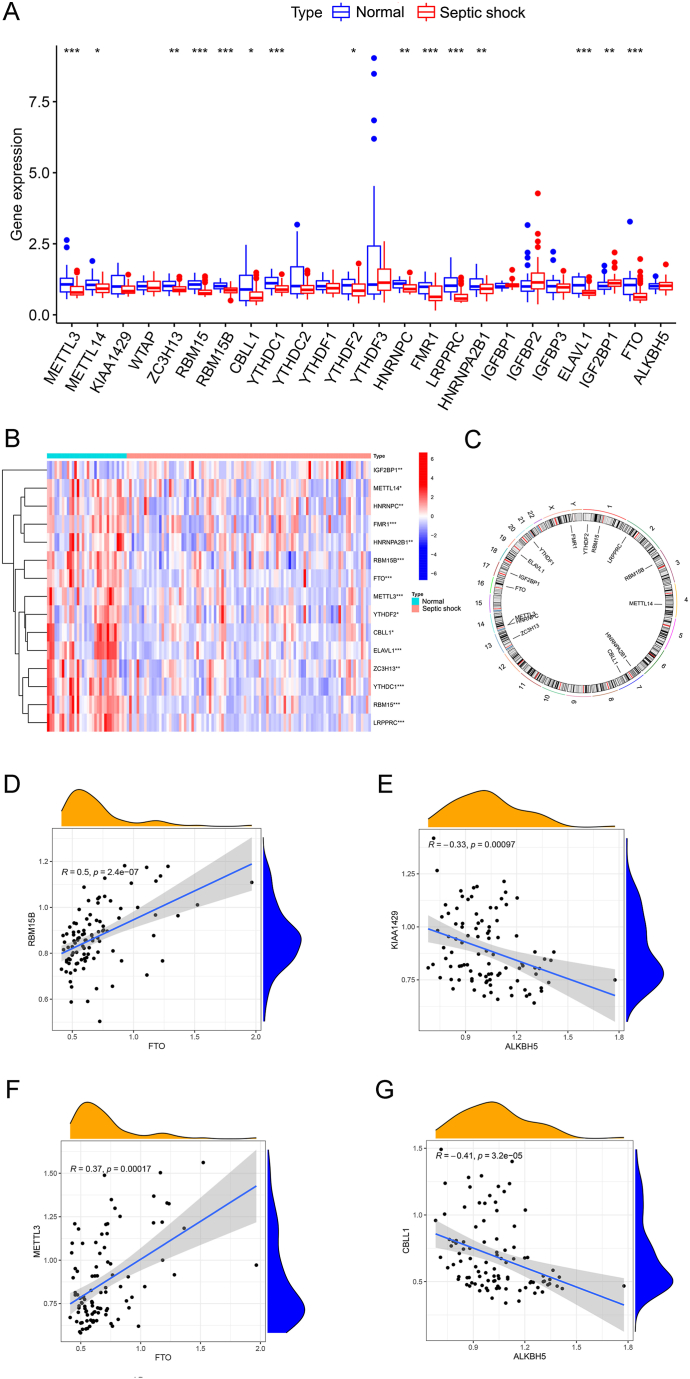
Differential expression and correlation analysis of m6A regulators. (A) Expression of m6A regulators in the two groups; (B) heatmap of differentially expressed genes; (C) locations of the 15 different m6A regulators on the corresponding chromosomes; (D–G) correlation between differentially expressed m6A regulators. ∗ *p* < 0:05, ∗∗ *p* < 0.01, ∗∗∗ *p* < 0.001.

The flow of dataset analysis is shown in Fig. 1. After comparing the septic shock group with the normal control group, 15 differentially expressed m6A regulators were identified, including METTL3, METTL14, ZC3H13, RBMl5, RBM15B, CBLL1, YTHDFl, YTHDF2, HNRNPC, FMR1, LRPPRC, HNRNPA2B1, ELAVL1, IGF2BP1, and FTO. Except for IGF2BP1, which was upregulated in the septic shock group, the expression of the remaining 14 genes was downregulated (Fig. 2A and B). The Rcircos R package was used to draw circular graphs. Information on the chromosomal locations of these 15 m6A regulators is shown in Fig. 2C.

Correlation analysis revealed strong positive correlations between FTO and both METTL3 and RBM15B and strong negative correlations between ALKBH5 and both KIAA1429 and CBLL1. The absolute values of the correlation coefficients were all above 0.3 (Fig. 2D–G). Although the roles of erasers and writers are opposite, the high expression of erasers did not necessarily imply a low expression of writers. This indicates that crosstalk may occur among the writers, readers, and erasers owing to the influence of complex mechanisms. This finding is consistent with those of previous studies [22].

### 3.3 Screening marker m6A regulators

**Fig. 3:**
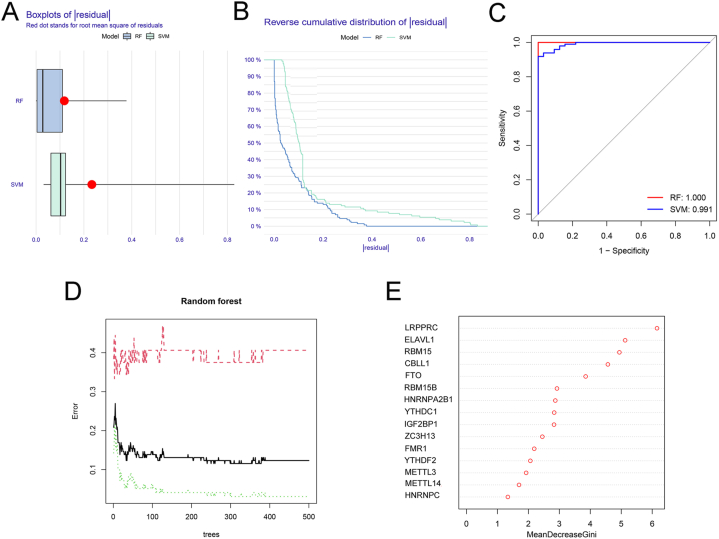
Machine learning to screen marker m6A regulators. (A) Boxplots of residual values of random forest (RF) and support vector machine (SVM) models. The red dot represents the mean residual value; (B) reverse cumulative distribution of residuals; (C) receiver operating characteristic curves showing that both the RF model and the SVM model were accurate; (D) the error value of the random forest. The red line represents the error of the septic shock group, the green line represents the error of the normal group, and the black line represents the total sample error; (E) mean decrease in Gini score of 15 differentially expressed m6A regulators. (For interpretation of the references to colour in this figure legend, the reader is referred to the Web version of this article.)

We first used the RF and SVM machine learning methods for the transcriptome data and the expression levels of the above 15 m6A genes were considered independent variables, and the normal and septic shock groups as the outcome variables. We analyzed the boxplots and cumulative residual distributions of both models to determine the one with the best performance. Fig. 3A shows that the mean residual value for the RF was lower than that for the SVM. The residual inverse cumulative distribution line of RF lay mostly within the residual line of the SVM (Fig. 3B), indicating that the difference between the predicted value of RF and the true value was smaller, and the model was more accurate. Similarly, ROC analysis suggested a higher AUC for RF (Fig. 3C). Therefore, the RF method was chosen to screen the m6A regulators of the disease signatures.

The number of trees corresponding to the point with the smallest cross-validation error was considered the optimum number. As shown in Fig. 3D, the cross-validation minimum error point for total sample error (black) was 0.115 for 240 optimal random forest trees. The importance scores of the m6A genes were further obtained using the RF model. The higher the mean decrease in the Gini score, the more important the gene is. The six m6A regulators with the highest significance scores were selected for subsequent analyses, namely LRPPRC, ELAVL1, RBM15, CBLL1, FTO, and RBM15B (Fig. 3E).

### 3.4 Constructing a risk prediction model for septic shock in children with marker m6A regulators

**Fig. 4:**
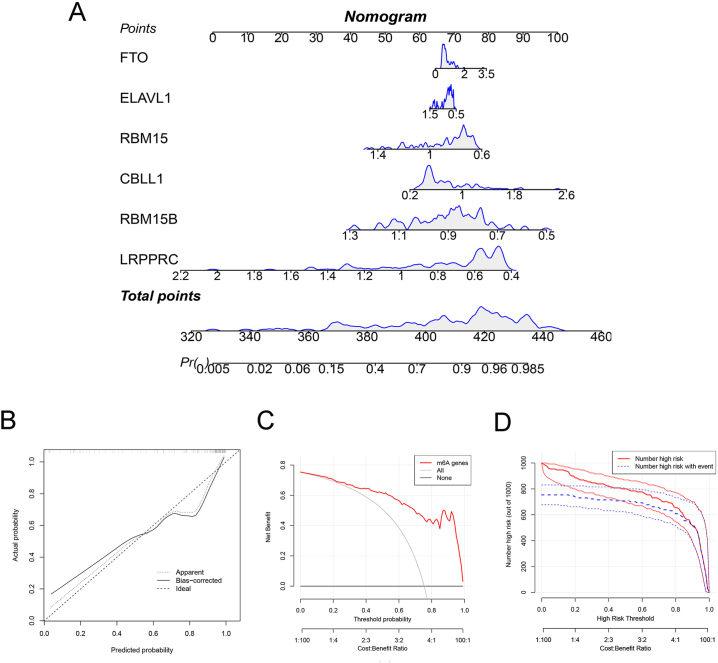
The risk prediction model for paediatric septic shock with m6A regulators. (A) The nomogram of the model; (B) the calibration curve; (C) the clinical decision curve; (D) the clinical impact curve for 1000 patients; the red line shows the total number who would be deemed at high risk for each risk threshold; the blue line shows true positives (cases). (For interpretation of the references to colour in this figure legend, the reader is referred to the Web version of this article.)

Using the six m6A regulatory markers, we constructed a risk prediction nomogram model for septic shock in children (Fig. 4). Calibration curves showed good agreement between the predicted and observed curves. The clinical decision curve showed a higher clinical net benefit for the prediction model curve than for the extreme curve across nearly all threshold probabilities. The clinical impact curve showed that the high-risk population judged by the prediction model as having septic shock was highly matched to the population that developed septic shock.

### 3.5 m6A molecular subtypes of paediatric septic shock

**Fig. 5:**
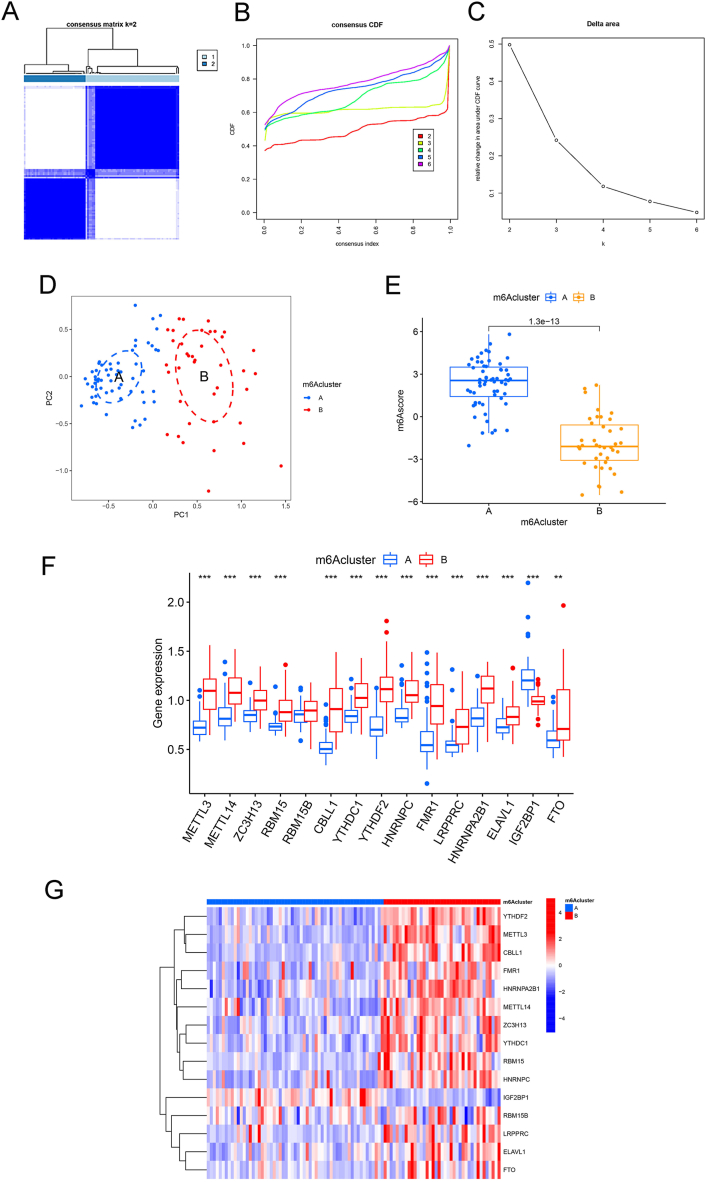
m6A molecular subtypes in paediatric septic shock. (A) Consensus matrix for k = 2; (B) CDF curves for k = 2–6; (C) relative change in the area under the CDF curves for k = 2–6. (D) principal component analysis for the two clusters; (E) differences in m6Ascore between subtypes; (F) differences in m6A regulators between subtypes; (G) heatmap of differentially expressed m6A regulators. ∗∗ *p* < 0.01, ∗∗∗ *p* < 0.001.

Fig. 5A shows a heatmap of the clustering matrix from k = 2 to k = 6. The darker the blue within the classification or the lighter the blue between the classifications, the better the classification effect. When k = 2, the blue inside the subtype was the darkest and the blue outside the subtype was the lightest. Fig. 5B shows the clustering scores from the CDF curve at different values of k. When the clustering score reached an approximate maximum, the clustering analysis results were considered the most reliable and the corresponding k value was the best, resulting in optimal classification. We considered the k value with the smallest declining gradient of the CDF. Fig. 5C shows the relative change in the area under the CDF curve between k and k-1. When the relative change in the area under the curve tended to stabilise, the optimal k was determined. Because of the absence of k = 1, it was impossible to evaluate the relative changes in the area under the curve at k = 2. After evaluating the discriminatory clarity of the consensus matrix heatmap, the clustering score from the CDF curve and the relative change in the area under the CDF curve, a binary cluster solution (k = 2) was selected. In Fig. 5D, each point in the PCA plot represents a sample, and the blue and red points represent the samples from clusters A and B, respectively. The samples from Clusters A and B were clearly separated rather than overlapping. This indicated that septic shock in children could be divided into two subtypes based on the differential expression of m6A regulators. Fifty-nine samples and 39 samples were classified in m6Acluster A and m6Acluster B, respectively.

Fig. 5E–G shows the differential expression analysis of the m6Ascore and m6A regulators between Clusters A and B. In Cluster A, except for RBM15B and IGF2BP1, the m6A regulators were expressed at higher levels than those in Cluster B. Additionally, the m6Ascore was also significantly higher in Cluster A than in Cluster B.

### 3.6 Heterogeneity of immune cell infiltration between m6A subtypes

**Fig. 6:**
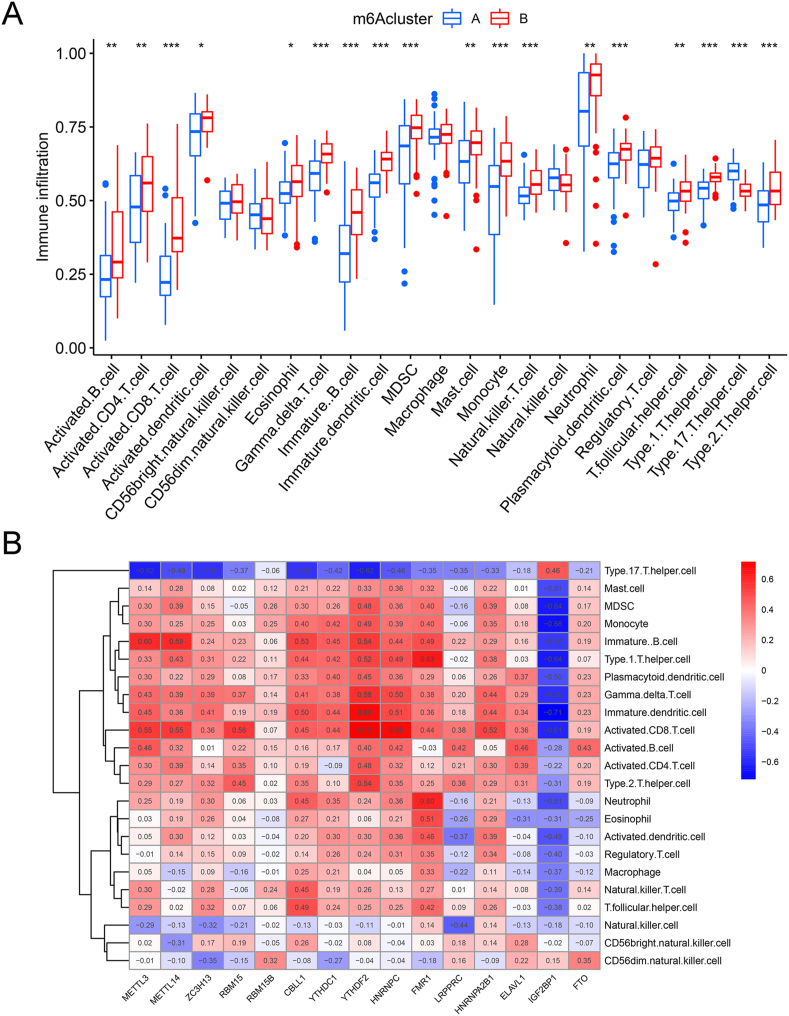
Heterogeneity in immune cell infiltration between m6A subtypes. (A) Different patterns of immune cell infiltration between Cluster A and B; (B) correlation analysis between m6A regulators and immune cell infiltration. ∗ *p* < 0:05, ∗∗ *p* < 0.01, ∗∗∗ *p* < 0.001.

After ssGSEA, obvious heterogeneity between subtypes' immune statuses was identified (Fig. 6A). The abundance of 18 immune cell types differed significantly between the two subtypes. The degree of infiltration of type 17 T helper cells in Cluster A was higher than that of the other 17 immune cells in Cluster B (Fig. 6A). Cluster A showed the downregulation of both pro-inflammatory and anti-inflammatory immune signatures, implying that the modification patterns have dual effects on inflammatory immunity.

Correlation analysis between m6A regulators and immune cell infiltration revealed a strong association (Fig. 6B). Among them, the most relevant to immune cells was IGF2BP1. Most immune cells showed higher levels of infiltration in the IGF2BP1 low expression group than in the IGF2BP1 high expression group (Supplementary Fig. 1).

### 3.7 Heterogeneity of biological functions between m6A subtypes

**Fig. 7:**
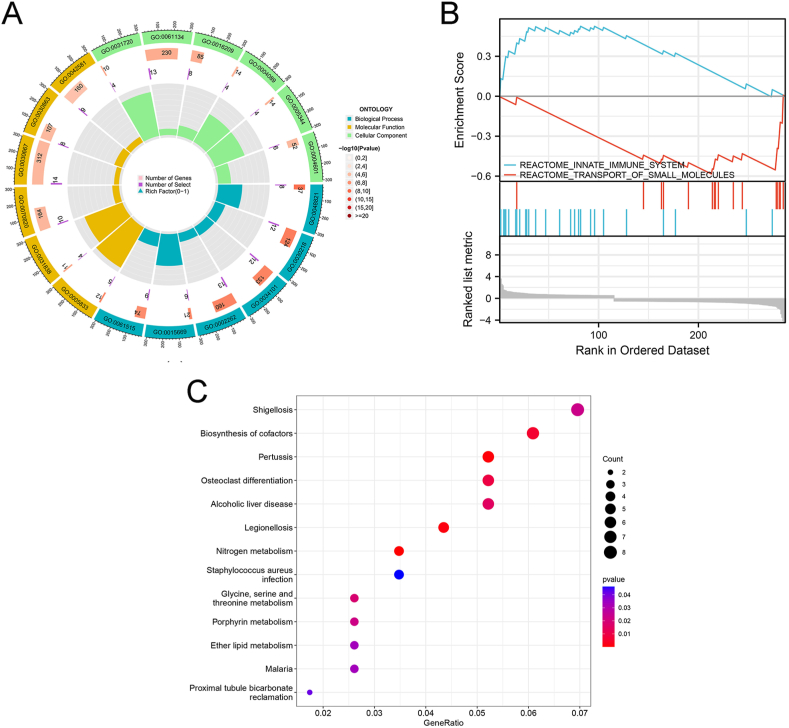
Heterogeneity in biological functions between m6A subtypes. (A) Gene Ontology analysis; (B) Gene Set Enrichment Analysis; (C) Kyoto Encyclopedia of Genes and Genomes analysis.

To explore the biological behaviour of these distinct m6A modification patterns, we performed an enrichment analysis. A total of 332 DEGs were selected from the expression matrices of different subtypes. The screened DEGs were subjected to functional enrichment analysis. The results of the GO analysis are shown in Fig. 7A and Supplementary Table 3. Enriched GO biological process terms included homeostasis of the number of cells, myeloid cell homeostasis, and positive regulation of the cell cycle process; cellular component relevant to DEGs were mainly associated with secretory granule membranes, specific granules, and tertiary granules; molecular function relevant to DEGs were mainly associated with oxygen carrier activity, antioxidant activity, and peroxidase activity.

KEGG analysis showed that enriched pathways relevant to DEGs mainly included biosynthesis of cofactors, osteoclast differentiation, and nitrogen metabolism (Fig. 7C and Supplementary Table 4).

GSEA-enriched pathways mainly involved the reactome innate immune system and reactome transport of small molecules (Fig. 7B and Supplementary Table 5).

### 3.8 Verification of selected key genes at the transcriptional level

**Fig. 8:**
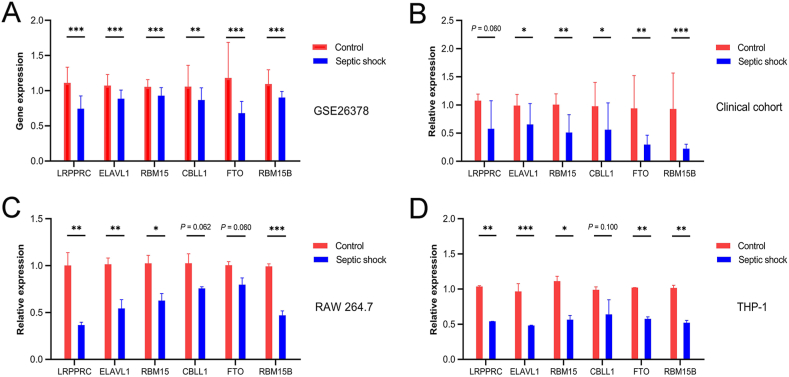
Validation of six marker m6A regulators. (A) Expression of six marker m6A regulators in GSE26378; (B) analysis of six marker m6A regulators in the clinical cohort by real-time quantitative PCR (RT-qPCR); (C) validation of six marker m6A regulators in RAW 264.7 cells by RT-qPCR; (D) validation of six marker m6A regulators in THP-1 cells by RT-qPCR. ∗ *p* < 0.05, ∗∗ *p* < 0.01, ∗∗∗ *p* < 0.001.

The GSE26378 dataset was used to validate the expression of the six m6A regulators (Fig. 8A). Additionally, RT-qPCR was used to compare the expression of six key genes between the disease and control groups in the clinical cohort (Fig. 8B), RAW 264.7 cells (Fig. 8C), and THP-1 cells (Fig. 8D). The expression of all six genes was consistent with the trend observed in microarray analysis.

## 4 Discussion

Septic shock is a common acute and critical illness in paediatric patients, with a high degree of heterogeneity, which largely contributes to treatment failure. Therefore, it is important to explore the molecular mechanism underlying the heterogeneity in children with septic shock to guide the treatment of the disease. m6A methylation is the most common form of mRNA modification which plays an important role in the formation of inflammation. The discovery of m6A opens up a new avenue for epigenetic studies and investigating inflammation-related diseases. At present, the study of m6A in children with septic shock is in the initial stages; however, in other immune-inflammatory diseases, m6A-related genes have been confirmed as potential diagnostic markers. Therefore, this study used machine-learning methods to identify m6A regulators in children with septic shock, providing new targets for the development of clinical molecular diagnostic markers and targeted therapeutic drugs in the future.

To the best of our knowledge, this is the first study on the transcriptome map of m6A regulators focusing on the landscape and function of m6A RNA modifications in paediatric septic shock. m6A regulators were found to be closely associated with the heterogeneity of septic shock in children. The risk-prediction model for septic shock based on m6A regulators has strong consistency and high clinical practicability. Based on the abnormal expression of m6A regulators, two subtypes of m6A were identified in septic shock. Significant differences were observed in RNA epigenetics, immune statuses, and biological processes between the two m6A subtypes. Finally, six marker m6A regulators were verified in additional samples.

m6A methylation is an epigenetic modification that occurs in most mammals [23]. m6A methylation is primarily regulated by methyltransferases (writers), demethylases (erasers) and, effector proteins (readers) [24]. RNA methylation occurs under the catalysis of the methyltransferase complex; demethylation is the removal of the methyl group by a demethylase to reverse m6A methylation. Effector proteins selectively recognise and bind to the m6A modification sites on RNA, thus regulating variable splicing, extranuclear transport, translation, and degradation of RNA, and ultimately regulating post-transcriptional gene expression. In the present study, six marker m6A regulators were identified, including LRPPRC, ELAVL1, RBM15, CBLL1, FTO, and RBM15B. We constructed a superior risk prediction model for septic shock in children using m6A regulators. Previous studies have identified LRPPRC and ELAVL1 as m6A readers, CBLL1, RBM15, and RBM15B as m6A writers, and FTO as an m6A eraser. The expression levels of the intracellular writer and eraser genes determine the methylation level of m6A, whereas the protein expressed by the reader can bind to m6A methylation sites to execute biological functions [25]. Although the mechanisms of action of these genes in children with septic shock remain unclear, some studies have confirmed that these six m6A regulators are associated with immune-inflammatory diseases [[26], [27], [28], [29], [30]]. Understanding the biological functions of these key genes in children with septic shock requires comprehensive in vivo and in vitro studies. The present study further revealed that these six m6A regulators play important roles in paediatric septic shock. The next step was to verify these m6A regulators experimentally and study their specific mechanisms in children with septic shock.

In this study, the mRNA transcriptome differences between different m6A modification patterns were found to be significantly associated with m6A and immune-related biological pathways. This study revealed a strong correlation between immune heterogeneity and m6A regulation in children with septic shock. Children with septic shock were classified according to the expression levels of m6A regulators. We found that the different subtypes have different immune states. The m6A modification pattern characterised by an immune-excluded phenotype exhibited a high m6Ascore, while the m6A modification pattern characterized by an immune-inflamed phenotype exhibited a low m6Ascore, consistent with the previous findings. This indicates that the m6Ascore is a reliable and powerful tool for comprehensively evaluating m6A modification patterns and can be used to determine the inflammatory immune infiltration pattern, that is, the inflammatory immune phenotype [22,31]. During the development of septic shock, hyperimmunity and immunosuppression are disadvantageous to the body [32]. However, owing to the immature immune system of children complex immune heterogeneity, and unknown mechanisms of septic shock, it is difficult to clearly describe an individual's immune status. Because the activity of the immune system is related to the expression of m6A regulators [33], the results of this study expand the application of the theory to children with septic shock.

The results of functional enrichment showed differences in the dynamic balance of cell numbers, redox reactions and innate immune system responses between the two subtypes. These factors are also closely associated with septic shock. Children, especially newborns, have an immature adaptive immune system. The struggle between innate immune cells and pathogenic microorganisms dominates the development of sepsis [34]. Shortly after the source of infection is detected, first-line defenders, including neutrophils, macrophages, and dendritic cells, begin to proliferate through recognition mechanisms to eliminate the infection as soon as possible [35]. During acute inflammatory response induced by septic shock, systemic immune cells undergo various phenotypic changes that directly affect their production, function, and survival. These changes can cause the organism to develop immunosuppression, followed by septic shock or persistent and recurrent secondary infections [36]. Simultaneously, the body produces large amounts of reactive oxygen species, leading to oxidative stress. Oxidative stress is the main factor leading to high mortality in many diseases such as septic shock [37]. Therefore, the innate immune response needs to achieve a balance in the number of immune cells along with maintaining good redox homeostasis. It is necessary to further explore the specific function of immune cells in different stages of septic shock and treatment strategies to restore the balance between the immune system and the redox response. This study deepens our understanding of the molecular characteristics and biological functions of different m6A subtypes associated with septic shock in children.

The strengths of this study are as follows: (1) the transcriptome data of a large sample were selected to identify the m6A regulators associated with septic shock. (2) For the selected m6A regulator markers in children with septic shock, qPCR experiments were performed using clinical and cellular samples for validation. The experimental results were consistent with the bioinformatics predictions. (3) This study differs from previous reports because we used machine learning methods to screen for m6A regulators associated with the occurrence of septic shock in children. When the feature space is large, the performance of logistic regression is poor. It is also prone to lack of fit and cannot handle a large number of multiclass variables well. However, this study had several limitations. First, there is a lack of in-depth research on the mechanism of m6A regulation, which is a future research direction. Second, because the dataset did not provide detailed clinical information for each patient, it was impossible to analyse the relationship between different molecular subtypes and other clinical parameters. Third, only a small number of clinical samples were used for validation. The next step is to collect more clinical samples and clinical information to establish a regression model.

## 5 Conclusions

In conclusion, this study has made the following contributions to the understanding of septic shock in children. This is the first study on the transcriptional profile of m6A regulators in children with septic shock, revealing that m6A methylation is crucially associated with the disease. Second, the septic shock risk prediction model based on the m6A regulator marker had strong consistency and high clinical practicability and could effectively distinguish children at risk of shock. It can be used to guide risk prediction for septic shock in children. Third, these findings strengthen the understanding of the molecular characteristics and specific immune status of subgroups of highly heterogeneous syndromes (septic shock in children) and provide a reference for precision medicine. As m6A RNA methylation is considered a reversible and adjustable process, drugs targeting m6A methylation may be new treatment options for paediatric septic shock.

## Author contribution statement

Huabin Wang: Conceived and designed the experiments; Performed the experiments; Analyzed and interpreted the data; Wrote the paper.

Junbin Huang: Analyzed and interpreted the data; Contributed reagents, materials, analysis tools or data.

Cheng Guo: Performed the experiments; Analyzed and interpreted the data.

Jingfang Wu; Liyuan Zhang:Contributed reagents, materials, analysis tools or data.

Xueyun Ren; Lijun Gan: Conceived and designed the experiments; Contributed reagents, materials, analysis tools or data; Wrote the paper.

## Data availability statement

Data associated with this study has been deposited at The datasets analyzed in this study could be found in GSE26440 at https://www.ncbi.nlm.nih.gov/geo/query/acc.cgi?acc=GSE26440, and in GSE26378 at https://www.ncbi.nlm.nih.gov/geo/query/acc.cgi?acc=GSE26378.

## Declaration of competing interest

The authors declare that they have no known competing financial interests or personal relationships that could have appeared to influence the work reported in this paper.
