# Supplementary material for: Molecular subtypes based on N6-methyladenosine RNA methylation demonstrate the heterogeneity of immune and biological functions in pediatric septic shock
Source: Heliyon. 2023 Oct 6;9(10):e20714. doi: 10.1016/j.heliyon.2023.e20714 (PMC10568115; doi:10.1016/j.heliyon.2023.e20714)
Supplement: Multimedia component 1 [file mmc1.docx]

Supplementary Table 1. Baseline characteristics of recruited datasets.

| Characteristic | GSE26440 | |
| --- | --- | --- |
|  | Septic shock | Control |
| Numbers | 98 | 32 |
| Age, median months (range) | 26 (0-131) | 20 (0-116) |
| Sex, n (%) |  |  |
| Male | 52 (53.1) | 19 (59.4) |
| Female | 46 (46.9) | 13 (40.6) |
| Co-morbidity, n (%) | 41 (41.8) | – |
| Immune suppression, n (%) | 23 (23.5) | – |
| Using hydrocortisone, n (%) | 35 (35.7) | – |
| Gram-positive bacteria, n (%) | 23 (23.5) | – |
| Gram-negative bacteria, n (%) | 20 (20.4) | – |
| Negative cultures, n (%) | 45 (45.9) | – |

Supplementary Table 2. The primer pairs used in qPCR.

| Primer name | Forward（5'-3'） | Reverse（5'-3'） |
| --- | --- | --- |
| LRPPRC | ATCATGGCGGAGAGATTGGC | AAATCGGGGTTTGTTCAGCA |
| ELAVL1 | TCGGGATAAAGTAGCAGGACAC | AGCGTGTTGATCGCTCTCTC |
| RBM15 | AGTAGACTTTGCCGACACCG | GTGGCACCCAATCAGAGTCA |
| CBLL1 | GCACCGCGAACTCAAAGAAC | TCAAATCCTTCTTCATCACCAGG |
| FTO | CTGCTCACTCCGGTATCTCG | GACCGTAAAGAGCCTGGTGT |
| RBM15B | GGGACCTAACACGTCACTGG | CTCATTTTAGCACAGGCGGC |
| GAPDH | GCACCGTCAAGGCTGAGAAC | TGGTGAAGACGCCAGTGGA |
| Lrpprc | GACCCGCGCGTTGGC | TCCCTTTTCTCAGCAACGATGG |
| Elavl1 | TGGGCGAATCATCAACTCCA | CGGATAAAGGCAACCCCTCT |
| Rbm15 | AAGTGGAGGACGGCATCTTG | TGCCCCGTCTATCTCTGACT |
| Cbll1 | TGCCACCAGACAAGCATCAT | ATGGACAATTCTGCCGGAGG |
| Fto | GACGTGGTGAGGATCCAAGG | AGCCTCTGTGTACTTGACCG |
| Rbm15b | ATTACGGGCTGTACGACGAC | ATTACGGGCTGTACGACGAC |
| Gapdh | TGTCTCCTGCGACTTCAACA | GGTGGTCCAGGGTTTCTTACT |

Supplementary Table 3. Results of Gene Ontology analysis.

| ONTOLOGY | ID | Description | GeneRatio | BgRatio | pvalue | p.adjust | qvalue |
| --- | --- | --- | --- | --- | --- | --- | --- |
| BP | GO:0048821 | erythrocyte development | 8/246 | 37/18800 | 2.13E-08 | 6.56E-05 | 6.09E-05 |
| BP | GO:0030218 | erythrocyte differentiation | 12/246 | 124/18800 | 8.41E-08 | 0.000129 | 0.00012 |
| BP | GO:0034101 | erythrocyte homeostasis | 12/246 | 133/18800 | 1.83E-07 | 0.000134 | 0.000124 |
| BP | GO:0002262 | myeloid cell homeostasis | 13/246 | 160/18800 | 1.92E-07 | 0.000134 | 0.000124 |
| BP | GO:0015669 | gas transport | 6/246 | 21/18800 | 2.17E-07 | 0.000134 | 0.000124 |
| BP | GO:0061515 | myeloid cell development | 9/246 | 74/18800 | 5.12E-07 | 0.000262 | 0.000244 |
| BP | GO:0048872 | homeostasis of number of cells | 15/246 | 282/18800 | 4.94E-06 | 0.002167 | 0.002014 |
| BP | GO:0090068 | positive regulation of cell cycle process | 13/246 | 247/18800 | 2.38E-05 | 0.00882 | 0.008197 |
| BP | GO:0019755 | one-carbon compound transport | 4/246 | 14/18800 | 2.58E-05 | 0.00882 | 0.008197 |
| BP | GO:0015671 | oxygen transport | 4/246 | 15/18800 | 3.49E-05 | 0.010714 | 0.009956 |
| BP | GO:0042744 | hydrogen peroxide catabolic process | 5/246 | 30/18800 | 4.02E-05 | 0.011223 | 0.010429 |
| BP | GO:0098869 | cellular oxidant detoxification | 8/246 | 100/18800 | 5.06E-05 | 0.012966 | 0.01205 |
| BP | GO:0044772 | mitotic cell cycle phase transition | 17/246 | 440/18800 | 7.31E-05 | 0.017276 | 0.016055 |
| BP | GO:0042743 | hydrogen peroxide metabolic process | 6/246 | 55/18800 | 8.01E-05 | 0.017574 | 0.016332 |
| BP | GO:0045787 | positive regulation of cell cycle | 14/246 | 329/18800 | 0.000117 | 0.024 | 0.022303 |
| BP | GO:1990748 | cellular detoxification | 8/246 | 115/18800 | 0.000136 | 0.026131 | 0.024284 |
| BP | GO:0006662 | glycerol ether metabolic process | 4/246 | 22/18800 | 0.000174 | 0.031425 | 0.029203 |
| BP | GO:0044839 | cell cycle G2/M phase transition | 9/246 | 155/18800 | 0.000208 | 0.033284 | 0.030931 |
| BP | GO:0030099 | myeloid cell differentiation | 15/246 | 391/18800 | 0.00021 | 0.033284 | 0.030931 |
| BP | GO:0097237 | cellular response to toxic substance | 8/246 | 123/18800 | 0.000217 | 0.033284 | 0.030931 |
| BP | GO:0007052 | mitotic spindle organization | 8/246 | 124/18800 | 0.000229 | 0.033509 | 0.03114 |
| BP | GO:0010389 | regulation of G2/M transition of mitotic cell cycle | 7/246 | 97/18800 | 0.000285 | 0.039797 | 0.036984 |
| BP | GO:1901990 | regulation of mitotic cell cycle phase transition | 13/246 | 321/18800 | 0.00033 | 0.044123 | 0.041004 |
| BP | GO:0018904 | ether metabolic process | 4/246 | 27/18800 | 0.000396 | 0.05073 | 0.047145 |
| BP | GO:0021670 | lateral ventricle development | 3/246 | 12/18800 | 0.000446 | 0.052072 | 0.048392 |
| BP | GO:0043312 | neutrophil degranulation | 3/246 | 12/18800 | 0.000446 | 0.052072 | 0.048392 |
| BP | GO:0010971 | positive regulation of G2/M transition of mitotic cell cycle | 4/246 | 28/18800 | 0.000458 | 0.052072 | 0.048392 |
| CC | GO:0005833 | hemoglobin complex | 5/255 | 12/19594 | 2.64E-07 | 9.66E-05 | 8.92E-05 |
| CC | GO:0031838 | haptoglobin-hemoglobin complex | 4/255 | 11/19594 | 8.61E-06 | 0.001575 | 0.001454 |
| CC | GO:0070820 | tertiary granule | 10/255 | 164/19594 | 5.93E-05 | 0.005798 | 0.005353 |
| CC | GO:0030667 | secretory granule membrane | 14/255 | 312/19594 | 6.35E-05 | 0.005798 | 0.005353 |
| CC | GO:0030863 | cortical cytoskeleton | 8/255 | 107/19594 | 7.92E-05 | 0.005798 | 0.005353 |
| CC | GO:0042581 | specific granule | 9/255 | 160/19594 | 0.000254 | 0.015513 | 0.014322 |
| CC | GO:0070821 | tertiary granule membrane | 6/255 | 73/19594 | 0.000376 | 0.019671 | 0.01816 |
| CC | GO:0000940 | outer kinetochore | 3/255 | 12/19594 | 0.000439 | 0.020101 | 0.018557 |
| CC | GO:0000775 | chromosome, centromeric region | 10/255 | 227/19594 | 0.000813 | 0.033068 | 0.030529 |
| CC | GO:0098687 | chromosomal region | 13/255 | 366/19594 | 0.001071 | 0.039185 | 0.036176 |
| MF | GO:0031720 | haptoglobin binding | 4/251 | 10/18410 | 6.64E-06 | 0.003142 | 0.002911 |
| MF | GO:0061134 | peptidase regulator activity | 13/251 | 230/18410 | 1.72E-05 | 0.003142 | 0.002911 |
| MF | GO:0016209 | antioxidant activity | 8/251 | 85/18410 | 2.08E-05 | 0.003142 | 0.002911 |
| MF | GO:0004089 | carbonate dehydratase activity | 4/251 | 14/18410 | 3.03E-05 | 0.003142 | 0.002911 |
| MF | GO:0005344 | oxygen carrier activity | 4/251 | 14/18410 | 3.03E-05 | 0.003142 | 0.002911 |
| MF | GO:0004601 | peroxidase activity | 6/251 | 52/18410 | 7.29E-05 | 0.00629 | 0.005828 |
| MF | GO:0016684 | oxidoreductase activity, acting on peroxide as acceptor | 6/251 | 56/18410 | 0.000111 | 0.008216 | 0.007613 |
| MF | GO:0051537 | 2 iron, 2 sulfur cluster binding | 4/251 | 24/18410 | 0.000289 | 0.015603 | 0.014459 |
| MF | GO:0051536 | iron-sulfur cluster binding | 6/251 | 67/18410 | 0.000301 | 0.015603 | 0.014459 |
| MF | GO:0051540 | metal cluster binding | 6/251 | 67/18410 | 0.000301 | 0.015603 | 0.014459 |
| MF | GO:0140104 | molecular carrier activity | 6/251 | 73/18410 | 0.00048 | 0.021712 | 0.020119 |
| MF | GO:0032396 | inhibitory MHC class I receptor activity | 3/251 | 12/18410 | 0.000503 | 0.021712 | 0.020119 |
| MF | GO:0030414 | peptidase inhibitor activity | 9/251 | 187/18410 | 0.001092 | 0.043494 | 0.040303 |
| MF | GO:0061135 | endopeptidase regulator activity | 9/251 | 194/18410 | 0.00141 | 0.051048 | 0.047303 |
| MF | GO:0032393 | MHC class I receptor activity | 3/251 | 17/18410 | 0.001478 | 0.051048 | 0.047303 |

BP, biological process; CC, cellular component; MF, molecular function.

Supplementary Table 4. Results of Kyoto Encyclopedia of Genes and Genomes analysis.

| ID | Description | GeneRatio | BgRatio | pvalue | p.adjust | qvalue |
| --- | --- | --- | --- | --- | --- | --- |
| hsa00910 | Nitrogen metabolism | 4/115 | 17/8165 | 7.72E-05 | 0.016433 | 0.016242 |
| hsa05133 | Pertussis | 6/115 | 76/8165 | 0.000672 | 0.071516 | 0.070685 |
| hsa05134 | Legionellosis | 5/115 | 57/8165 | 0.001187 | 0.084247 | 0.083269 |
| hsa01240 | Biosynthesis of cofactors | 7/115 | 153/8165 | 0.005729 | 0.305082 | 0.301539 |
| hsa04380 | Osteoclast differentiation | 6/115 | 128/8165 | 0.009242 | 0.393691 | 0.389119 |
| hsa04936 | Alcoholic liver disease | 6/115 | 142/8165 | 0.014893 | 0.52869 | 0.52255 |
| hsa00260 | Glycine, serine and threonine metabolism | 3/115 | 40/8165 | 0.018423 | 0.545052 | 0.538722 |
| hsa00860 | Porphyrin metabolism | 3/115 | 43/8165 | 0.022325 | 0.545052 | 0.538722 |
| hsa05131 | Shigellosis | 8/115 | 247/8165 | 0.02303 | 0.545052 | 0.538722 |
| hsa00565 | Ether lipid metabolism | 3/115 | 50/8165 | 0.033046 | 0.639889 | 0.632458 |
| hsa05144 | Malaria | 3/115 | 50/8165 | 0.033046 | 0.639889 | 0.632458 |
| hsa04964 | Proximal tubule bicarbonate reclamation | 2/115 | 23/8165 | 0.041042 | 0.728501 | 0.720041 |
| hsa05150 | Staphylococcus aureus infection | 4/115 | 96/8165 | 0.046195 | 0.756884 | 0.748094 |

Supplementary Table 5. Results of gene set enrichment analysis analysis.

| Description | setSize | enrichmentScore | NES | pvalue | p.adjust | qvalues | rank |
| --- | --- | --- | --- | --- | --- | --- | --- |
| REACTOME_INNATE_IMMUNE_SYSTEM | 26 | 0.525146 | 2.195834 | 0.002639 | 0.035948 | 0.02408 | 82 |
| REACTOME_TRANSPORT_OF_SMALL_MOLECULES | 16 | -0.58361 | -2.06298 | 0.003268 | 0.035948 | 0.02408 | 75 |
| NABA_MATRISOME | 16 | 0.639271 | 2.320589 | 0.005115 | 0.037511 | 0.025127 | 3 |
| NABA_MATRISOME_ASSOCIATED | 13 | 0.637685 | 2.206625 | 0.007335 | 0.040342 | 0.027024 | 2 |
| REACTOME_CELL_CYCLE | 15 | 0.598216 | 2.131745 | 0.010204 | 0.044825 | 0.030026 | 58 |
| REACTOME_CELL_CYCLE_MITOTIC | 13 | 0.617116 | 2.135451 | 0.012225 | 0.044825 | 0.030026 | 87 |
| REACTOME_NEUTROPHIL_DEGRANULATION | 19 | 0.527474 | 1.993253 | 0.018568 | 0.058355 | 0.03909 | 82 |
| REACTOME_RNA_POLYMERASE_II_TRANSCRIPTION | 20 | 0.466584 | 1.797841 | 0.048 | 0.132 | 0.088421 | 139 |
| REACTOME_INFECTIOUS_DISEASE | 13 | 0.483767 | 1.674011 | 0.0978 | 0.239065 | 0.16014 | 79 |


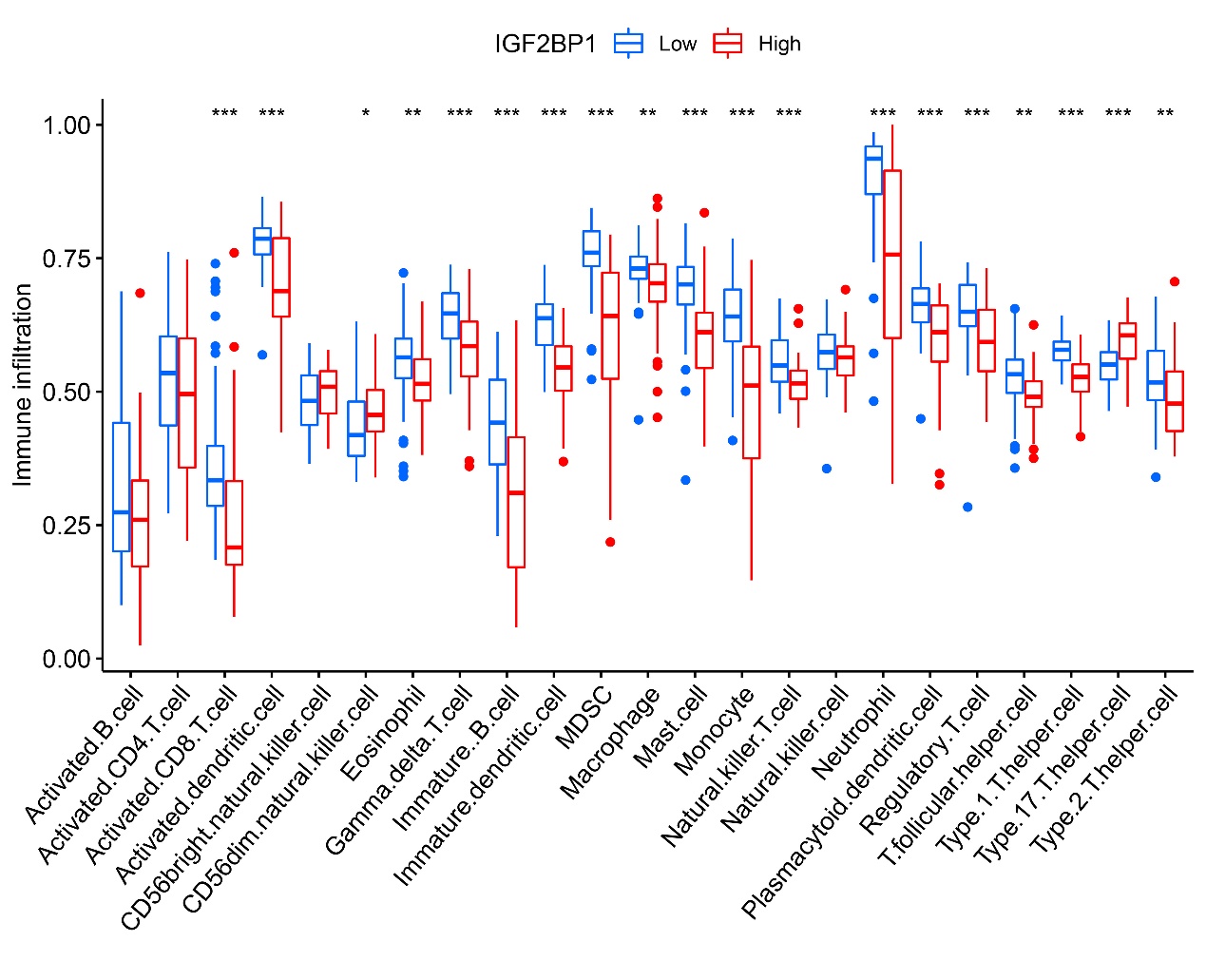


Supplementary Figure 1. Immune cell infiltration was compared between IGF2BP1 high and low expression groups. ^∗^*p* < 0:05, ^∗∗^*p* < 0.01, ^∗∗∗^*p* < 0.001.
